# Cylindroma, an uncommon presentation on ^18^F-DCFPyL PET/CT

**DOI:** 10.1007/s00259-020-04943-3

**Published:** 2020-07-11

**Authors:** B. P. F. Koene, Y. J. L. Bodar, D. Meijer, E. H. Jaspars, A. N. Vis, D. E. Oprea-Lager

**Affiliations:** 1 Department of Urology, Amsterdam University Medical Centers (VU University), Amsterdam, The Netherlands; 2 Department of Radiology & Nuclear Medicine, Amsterdam University Medical Centers (VU University), Amsterdam, The Netherlands; 3 Prostate Cancer Network, Amsterdam, The Netherlands; 4 Department of Pathology, Amsterdam University Medical Centers, Amsterdam, The Netherlands

A 68-year-old man presented with a persistently high prostate-specific antigen (PSA) level of 27.8 ng/mL. Subsequent multiparametric magnetic resonance imaging (mpMRI) classified a peripheral lesion in the left prostate lobe as PI-RADS II, and repeated systematic prostate biopsies were negative for cancer. Given his elevated PSA level, ^18^F-DCFPyL PET/CT was performed (327 MBq, 120 min after intravenous radiotracer injection).

Moderately increased PSMA expression was observed dorsally, in the peripheral zone of the prostate (left lobe (mid gland, SUV_max_ 5.61; PSMA-RADS, 4) and right lobe (mid gland, SUV_max_ 4.98; PSMA-RADS, 4)) on sagittal low-dose CT (A), sagittal PET attenuation correction image (B) and transversal images of PET (C), fused PET/low-dose CT (E), and low-dose CT (G), whereas diffuse, highly increased PSMA expression (SUV_max_ 6.86) was observed in a subcutaneous, well-circumscribed, nonencapsulated interscapular lesion of 20 × 14 mm (level thoracic vertebra 3–4), without infiltration of the subcutaneous fat (D, F, and H). Because of the elevated PSMA expression and the atypical location for a possible prostate cancer metastasis [1], histopathological evaluation of this lesion was indicated.

Histological examination revealed an epithelial tumor with the histopathological characteristics of a cylindroma: a benign tumor of the sweat glands. Cylindromas represent rare entities which may present sporadically or in a familial inherited form. They are usually (> 90%) located on the scalp or face, occur 9 times more in females than in males, and exceptionally may undergo malignant transformation. The preferred treatment of cylindromas is excision, being usually curative and preventing patients from inconveniencies due to location and, very rarely, from chance of malignant transformation. Subfigures I and J show the multilobulated tumor deep in the skin at magnification × 40 and × 400, the latter demonstrating the characteristic pattern of small circumscribed nests of neoplastic epithelial cells surrounded by a broad band of hyaline matrix in a “jigsaw-like pattern” [2, 3]. The tumor cells of the cylindroma were entirely negative in an ancillary immunohistochemical staining for PSA, making any association with prostate (cancer) tissue highly unlikely. The patient underwent transrectal PSMA-guided targeted biopsies of the prostate, which again showed no malignancy. Interestingly, the tumor cells of the cylindroma showed cytoplasmic expression of PSMA, as was demonstrated by immunohistochemical staining (subfigure K, antibody Dako Clone 3E6), which can explain the PET scan results.

Although PSMA uptake by PET imaging has been demonstrated in several non-prostatic diseases [1, 4, 5], this is the first reported case of a benign sweat gland tumor that reveals this characteristic. This is another argument for carefully interpreting radiolabeled PSMA PET/CT scans in oncology, together with the metastatic pattern, the grade of PSMA expression, the aspect on the CT, and the atypical location of avid lesions, in order to exclude potential pitfalls and to allow adequate therapy management.


